# The economic burden of bipolar disorder: a case study in Southern Iran

**DOI:** 10.1186/s12962-024-00560-1

**Published:** 2024-07-18

**Authors:** Zohreh Shaker, Zahra Goudarzi, Ramin Ravangard, Zinab Shaker, Arvin Hedayati, Khosro Keshavarz

**Affiliations:** 1 Student Research Committee, School of Health Management and Information Sciences, Shiraz University of Medical Sciences, Shiraz, Iran; 2 Health Human Resources Research Center, School of Health Management and Information Sciences, Shiraz University of Medical Sciences, Shiraz, Iran; 3 Faculty of Management and Medical Information Sciences, Kerman University of Medical Sciences, Kerman, Iran; 4 Department of Psychiatry, School of Medicine, Research Center for Psychiatry and Behavior Science, Hafez Hospital, Ebnesina Hospital, Shiraz University of Medical Sciences, Shiraz, Iran; 5 Emergency Medicine Research Center, Shiraz University of Medical Sciences, Shiraz, Iran

**Keywords:** Economic Burden, Bipolar disorder, Direct Medical cost, Direct non-medical cost, Indirect cost

## Abstract

**Background:**

Bipolar Disorder (BD) imposes considerable economic and social burdens on the community. Therefore, the present study aimed to determine the economic burden of bipolar disorder in patients referred to single-specialty psychiatric hospitals at the secondary and tertiary care level in 2022.

**Methods:**

This partial economic evaluation was conducted as a cross-sectional study in the south of Iran in 2022, and 916 patients were selected through the census method. The prevalence-based and bottom-up approaches were used to collect cost information and calculate the costs, respectively. The data on Direct Medical Costs (DMC), Direct Non-Medical Costs (DNMC), and Indirect costs (IC) were obtained using the information from the patients’ medical records and bills as well as the self-reports by the patients or their companions. The human capital approach was also used to calculate IC.

**Findings:**

: The results showed that in 2022, the annual cost of bipolar disorder was $4,227 per patient. The largest share of the costs was that of DMC (77.66%), with hoteling and ordinary beds accounting for the highest expenses (55.40%). The shares of DNMC and IC were 6.37% and 15.97%, respectively, and the economic burden of the disease in the country was estimated at $2,799,787,266 as well.

**Conclusion:**

In general, the costs of bipolar disorder treatment could impose a heavy economic burden on the community, the health system, the insurance system, and the patients themselves. Considering the high costs of hoteling and ordinary beds, it is suggested that hospitalization of BD patients be reduced by managing treatment solutions along with prevention methods to reduce the economic burden of this disease. Furthermore, in order to reduce the costs, proper and fair distribution of psychiatrists and psychiatric beds as well as expansion of home care services and use of the Internet and virtual technologies to follow up the treatment of these patients are recommended.

## Introduction

Bipolar Disorder (BD) is a type of brain disorder that causes extreme mood swings as well as changes in the body’s energy level and individual behaviors. People with bipolar disorder experience intense emotional states [1, 2]. In other words, bipolar disorder is a lifelong periodic mental illness; this chronic health condition is associated with periods of mania, hypomania, and major depressive disorder [3].

In some BD patients, the symptoms are so severe that they experience significant problems in doing their daily activities, including daily routines as well as work tasks or school assignments, and their behaviors in the community are severely affected by the disease [4]. Furthermore, according to a study conducted in 2020, people with BD suffer from financial, social, or occupational problems due to the disease, and impose a considerable socioeconomic burden on their communities and families [5].

In addition to the fact that this disease may lead to functional and cognitive disorders and reduce the quality of life, it also imposes a great economic burden on the community [6, 7]. The global prevalence of bipolar disease was 0.53% on average in 2019, with more than 39 million people suffering from the disease in the world [8]. The symptoms of this disease mostly start between the ages of 20 and 25 [9]. BD seems to be more common among women [10]. The lifetime prevalence of this disease and the prevalence for the age group of 18–65 years in 2019 in Iran were 0.92% and 1.1%, respectively, which were higher than the global average rates [8].

According to a study conducted in 2022, the annual economic burden of BD treatment in England was calculated at 6.43 billion pounds [11]. Similarly, another study conducted in the United States in 2015 showed that Direct Medical Costs (DMC) and Direct Non-Medical Costs (DNMC) were 23% and 5%, respectively (accounting for 28% of the costs altogether), and the rest was related to Indirect Costs (IC). The total cost of BD in 2015 was estimated at $202.1 billion as well [12].

In 2013, Bipolar Disorder was one of the 20 main causes of disability worldwide, leading to significant costs for communities [13]. According to what was mentioned above, health costs have increased significantly in recent decades around the world. Iran’s health system has also faced significant challenges due to the increased health costs. Considering the limited resources for providing health care services, the provision of effective services is one of the general concerns of health care systems around the world [14]. Cost-of-illness studies measure the economic burden of diseases [15].This type of study is used as a model to evaluate different therapeutic interventions and is also used in setting priorities for research in the field of health [16]. Cost-of-illness studies are common in health systems and are widely used by some organizations, including the World Health Organization and national health institutions [17].

As found out by reviewing the literature, related domestic studies were very few and to date, no study had been conducted on the economic burden of BD in Iran. According to the findings of the studies, the high prevalence of this disorder and the significant burden it imposed on individuals, the health system, and the community, multiplied the necessity of conducting the present study. Therefore, this research was conducted in order to determine the economic burden of bipolar disorder in Iran in 2022.

## Materials and methods

This is a partial economic evaluation study of the cost-of-illness type conducted as cross-sectional research in 2022 in Fars province. The province is located in the south of Iran with a population of over 5 million people (More than %5 population of Iran resides in Fars province). It has 55 public hospitals affiliated with Shiraz, Fasa, Jahrom, Larestan, and Gerash Universities of Medical Sciences. Two hospitals, i.e. Professor Moharari Hospital and Ibn-e Sina Hospitals, are single-specialty and work in the field of psychiatric disorders. We considered secondary and tertiary care levels.

The referral system for mental health services in Iran is one of the strategies for the prevention and treatment of mental disorders. Patients with mental problems are visited by a family physician or a health liaison, and if needed, they are referred to a specialist doctor or psychiatric service center [18]. The study population included all bipolar disorder patients who were referred and admitted to these two hospitals in 2022. Psychiatrists have diagnosed patients with bipolar disorder with the help of the Diagnostic and Statistical Manual of Mental Disorders (DSM-5) and the International Classification of Diseases 11th Revision (ICD-11), which are two major diagnostic manuals. According to the statistics of these centers, a total of 916 patients were studied through the census method.

The study protocol was approved by the Ethics Committee in November 2022 (code: IR.SUMS.NUMIMG.REC.1401.063). Psychiatrists have diagnosed patients with bipolar disorder with the help of the Diagnostic and Statistical Manual of Mental Disorders (DSM-5) which is a major diagnostic manual. All participants were informed, both verbally and in writing, of their right to withdraw from the study at any time. The informed consent was obtained from all subjects and their legal guardian(s) to participate in the study.

In addition, the costs were collected using a researcher-made cost collection form as well as the opinions of psychiatrists and health economists and the patients’ self-reports. The data collection form included four sections as follows: the patients’ demographic characteristics (age, sex, marital status, education level, employment status, type of basic insurance coverage, number of hospitalizations, residence, and average monthly income), and the information on Direct Medical Costs (DMC), Direct Non-Medical Costs (DNMC), and Indirect Costs (IC). This study was conducted from a community perspective; thus, all costs, including DMC, DNMC, and IC, were taken into consideration. To increase the accuracy of the cost data, the researchers tried to consider the opinions of the patients, their companions, and the psychiatric specialists. Furthermore, to avoid recall errors in IC and DNMC, the Outpatient information of patients for the 3 months was separated (The 3-month period of assessment of the patient in euthymic mood and after hospitalization was related, and the patient was in a stable condition.) and then multiplied by 4 to estimate the total annual cost. We have asked the patients and their companions about the number and cost of psychiatrist visits after discharge from the hospital and the number and cost of medications taken by the patient after discharge from the hospital.

Face-to-face or telephone interviews were conducted with the patients or their companions with the help of 4 members (the interviewers were two masters in health economics and two associate professors in health economics). Before the interview, the purpose of the research, the data collection form, and the interview method were explained to the interviewers. Interviews were taken from the patients, but some patients were not able to answer, so we asked the patients’ companions. The required data were collected from December 15, 2022, to February 28, 2023.

### Inclusion and exclusion criteria

As suggested by the experts and based on previous studies, the patients’ age was considered 18 to 65 years [19, 20]. In addition, those who had only been referred as outpatients were not included in the study due to the lack of medical records, and admitted patients were taken for this study. The exclusion criteria were as follows: Bipolar patients with co-occurring psychiatric illnesses, mental disability along with bipolar disorder, having been affected by BD through head trauma, lack of consent to participate in the study, and unwillingness to continue participation in the study according to the consent form.

### Measurement of direct medical cost

Each patient’s DMC was collected retrospectively using a researcher-made collection form by referring to the reference hospitals. The costs included visits, drugs, tests, radiology, concussion, hoteling services, etc., which were separately determined and extracted for inpatient services from the medical and financial records of the patients as well as the hospital information system in 2022 to increase the accuracy of the data. For outpatient services, they were collected through interviews with psychiatric specialists and the patients.

### Calculation of direct medical cost

The average annual DMC per patient = (average number of visits × visit tariff) + (average number of each medical service × tariff for each medical service) + (average number of each diagnostic service × tariff of each diagnostic service) + (average number of hospitalizations × tariff for each day of hospitalization) + (cost of each drug dose × number of prescriptions during a treatment period) [21].

All inpatient services were based on public tariffs. However, for follow-up visits and diagnostic services, patients use both private and public services, which according to the Ministry of Health reports, this ratio is 65% from the private sector and 35% from the public sector. It is worth noting that the percentage of medical care used was taken into account when calculating each case under the supervision of specialists. In addition to drugs (including tablets and injections), ECT therapy is also included in DMC. Services received due to drug side effects and the susceptibility of bipolar disorder patients to diseases such as cardiovascular diseases, thyroid issues, diabetes, and ovarian cysts have also been considered [22].

### Calculation of direct non-medical cost

The DNMC was obtained using a cost collection form and through patient interviews. It included travel expenses to medical centers to receive medical services, as well as accommodation and food expenses for the patients and their companions.

The average annual DNMC per BD patient was calculated as follows:

“Average cost per patient = number of visits to receive medical services per year × cost of each visit”.

### Measurement and calculation of indirect cost

The IC included absenteeism and reduced productivity of the patients and their companions, which was calculated through interviews with the patients and their companions based on their average daily income loss due to absence from work for hospitalization or treatment follow-up and the average daily income lost of each patient companion due to absence from work to accompany or care for the patient. The calculation process was based on the human capital approach [23]. In the present study, the patients’ wages were used to calculate the income lost. The minimum daily wage determined by the Ministry of Labor for housewives and students aged 15 to 65 ($33 considered as the average daily wage in 2022 for eight hours equivalent to one working day) was used as well [24].

Therefore, in this study, the IC was calculated using a checklist as well as asking the patients and their companions, and based on their monthly income. The human capital approach was also applied [23, 25].

### Economic burden imposed on all patients with bipolar disorder

In the present study, prevalence-based and bottom-up approaches were used to calculate the economic burden and costs. The former measured patient costs over a period of usually one year and the latter measured the resources used by any individual. Thus, the bottom-up approach was able to distinguish treatment differences between the patients [26]. The economic burden of the disease was calculated using the following formula: [21]

Economic burden∶ Total cost (Direct Medical Cost + Direct Non-medical Cost + Indirect Cost) × estimated number of patients aged 18–65 years in Iran.

It should be noted that in this research, all the costs were converted using the Rial rate and the reference currency index as each dollar was equivalent to 42,000 Rials in 2022 [27].

### Estimation of prevalence

According to the statistics of the World Bank in 2021, 68% of the Iranian population was 18 to 65 years of age [28]. Besides, the United Nations had declared the population of Iran to be 88,550,570 people in 2022 [29], and considering the prevalence of this disease in Iran (1.1%) [8], it was expected that there would be 662,358 BC patients in this age range. The Excel 2016 software was used for data analysis.

### Sensitivity analysis

To ensure the robustness of the results, the one-way sensitivity analysis was used in this study. For this purpose, the prevalence rate of bipolar disorder in Iran was considered variable according to the low and high prevalence limits reported by Global Health Data (GHD) (0.0088-0.0132) [8], and the disease population and the economic burden caused by it were calculated in the determined range. Furthermore, the cost components per patient were assumed constant.

## Results

### Demographic characteristics

A total of 916 patients participated in the study, whose demographic characteristics are presented in Table 1. The results showed that 67.03% of the patients were male. Besides, 40.93% of the patients were 18–34 years old, 47.81% were married, and 91.01% had a high school education level or lower. Of all the patients, 63.64% were unemployed, 93.77% were natives of Fars province, and 98.21% had insurance coverage. The average length of hospitalization was 25.05 days.

**Table 1:** Demographic characteristics of the patients with Bipolar Disorder in southern Iran in 2022

Variable		Number patients	%
sex	Male	615	67.03
	Female	301	32.97
Age	18–34	375	40.93
	35–49	353	38.55
	50–65	188	20.53
Marital status	Single	348	37.89
	Married	433	47.81
	Divorced	135	14.31
Education level	Secondary school or lower	827	91.01
	Academic	89	8.99
Employment status	Employed	298	32.69
	Retired	26	2.68
	Unemployed	592	64.63
Residence	Fars Province	856	93.77
	Other provinces	60	6.23
Basic insurance	Insured	900	98.21
	No insurance	16	1.79

### Direct and indirect costs

Table 2 shows the average direct and indirect costs of the patients with bipolar disorder. The average DMC for each patient was $3,281, and the cost of hoteling and bed was $1,819, accounting for the largest share of DMC (55.40%). The average DNMC for each patient was $271, the highest share of which was the cost of patient companions’ travel (47.92%). Finally, the average IC for each patient was $675, the largest share of which was related to patient absenteeism (84.31%).

**Table 2:** Mean direct medical, direct non-medical, and indirect costs per patient with bipolar disorder in 2022 from a social perspective

Type of cost		USD	%	Total cost (%)
Direct medical costs	Hoteling	1819		77.66
	Visit	704		
	Consultation and Psychology services	68		
	Nursing services	107		
	Diagnostic and Rehabilitation	118		
	Medicine and Consumables	196		
	Other services*	269		
	Total	3281		
Direct non-medical costs	Patients Transportation	97	35.99	6.37
	Patients’ commute Transportation	129	47.92	
	Patients’ accommodation and food	20	7.08	
	accommodation and food for the patients’ commute	25	9.01	
	Total	271	100	
Indirect costs	Missed workdays due to illness	571	84.31	15.97
	patient companions’ missed workdays due to patient care	104	15.69	
	Total	675	100	
costs	Total cost	4227	100	100

* Emergency and other services that have been received due to the side effects of drugs and also due to the susceptibility of bipolar disorder patients to diseases such as cardiovascular diseases, thyroid, diabetes, and ovarian cysts

### Economic burden of costs for BD in Iran

**Fig. 1:**
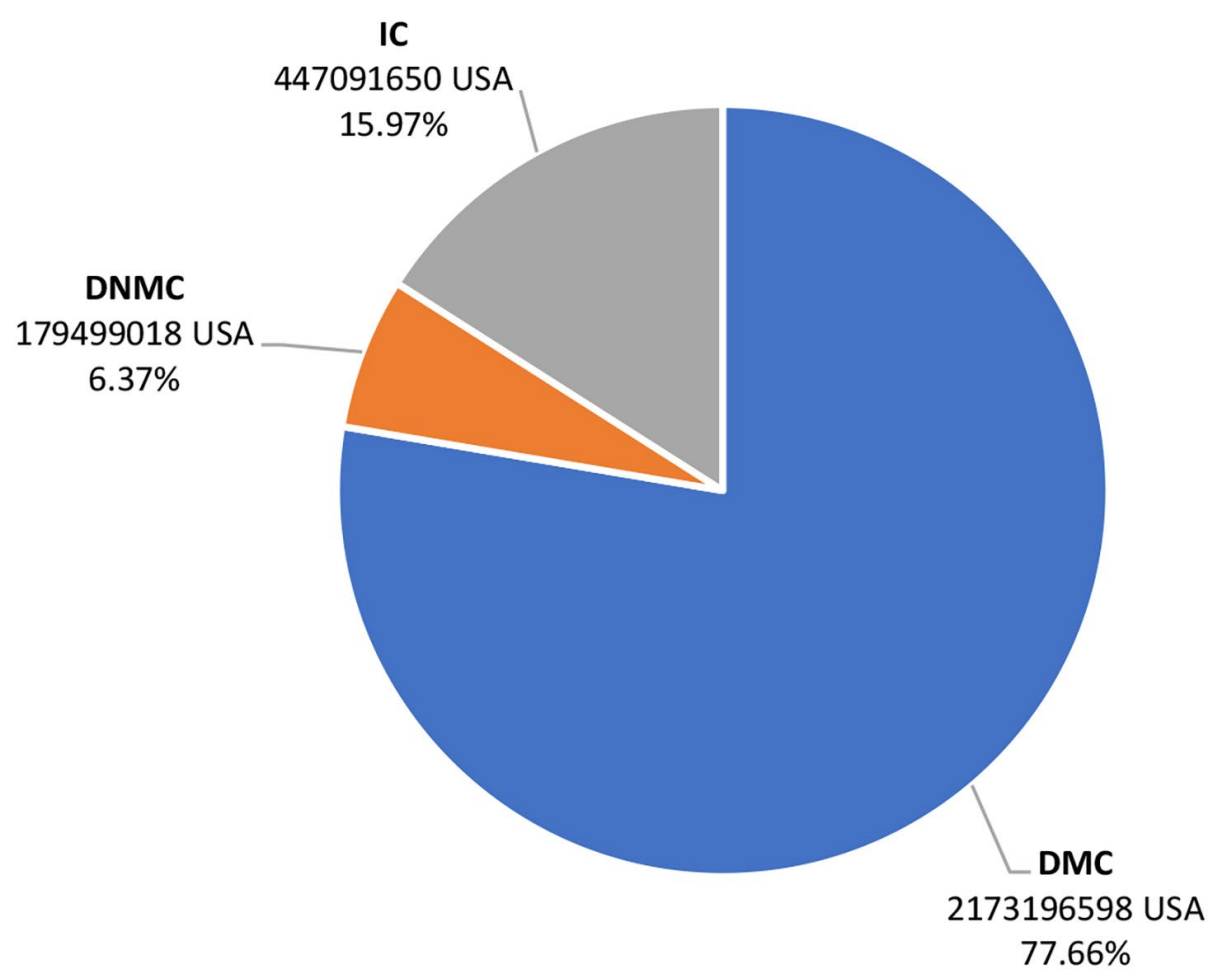
Estimation of annual economic burden of patients with bipolar disorder in Iran in 2022 from a social perspective (USD)

The economic burden of BC costs in Iran was estimated according to the number of patients in the country based on the average prevalence of the disease among people aged 18 to 65. The costs extracted from the results of this study and the estimated economic burden for all bipolar disorder patients in Iran are presented in Table 3. It was found that the total annual cost of BC patients in Iran was $2,799,787,266 in 2022. The annual cost of this disease per patient was $4,227 as well. The results also showed that DMC accounted for the largest share of the economic burden of bipolar disorder in the country with 77.66%. Figure 1 shows the average total costs of DMC, DNMC, and IC in Iran in 2022.

**Table 3:** Estimation of total annual costs of patients with Bipolar Disorder in 2022

Number of patients	Direct medical costs (USD)	Direct non-medical costs (USD)	Indirect costs (USD)	Economic burden (USD)
662,358	2,173,196,598	179,499,018	447,091,650	2,799,787,266
%	77.66	6.37	15.97	100

### Sensitivity analysis

The results of the one-way sensitivity analysis are presented in Table 4. Considering the changes in the prevalence rate, the total economic burden of bipolar disorder in Iran would vary from $1,530,460,384 to $2,295,689,132.

**Table 4:** Sensitivity analysis for DMCs, DNMCs, ICs, and total costs of BD patients in Iran in 2022

One-way Sensitivity analysis (USD)	Number of patients in Iran	DMC (USD)	DNMC (USD)	IC (USD)	COI (USD)
**Upper limit**	794,830	1,782,780,905	146,138,258	366,769,970	2,295,689,132
**Lower limit**	529,887	1,188,521,351	97,425,566	244,513,467	1,530,460,384

## Discussion

The increased costs of health systems around the world, especially in low- and middle-income countries like Iran, have become a major concern for health managers and policymakers. The main reason is the increase in the number of non-communicable and chronic diseases, such as bipolar disorder, whose treatment costs have increased rapidly in recent years [14, 30, 31]. This study aimed to calculate the costs of bipolar disorder for the patients referred to the reference medical centers in southern Iran and to determine the economic burden of this disease in the country in 2022.

The demographic characteristics in this study showed that most of the patients were male, in the age range of 18–34 years, married, with an education level below the high school diploma, unemployed, covered by a health insurance organization, living in the cities of Fars province, and without any income. The results of the study by Kheradmand et al. (2022) in Iran on BD patients also showed that most of the patients (52.5%) were male [32]. In their study, Amini et al.(2010) in Iran [33] showed that the mean age of the patients was 32.2 years, which is consistent with the results of the present study.

The results of this study showed that bipolar disorder imposed a significant economic burden on the health system and the community. The economic burden of this disease in the country was estimated to be $2,799,787,266, with about 5% of the total budget of the health system in 2022 allocated to it. Considering DMC, DNMC, and IC, it was found that DMC was the highest (77.66%), of which hoteling and bed (55.40%) had the largest share. The reason for the high cost of hoteling could be the high cost of beds and the services provided by the service providers to the studied patients as well as the high average number of their hospitalization days. In most previous studies, the costs of hospitalization and hoteling services had the largest share of DMC [6, 19, 34, 35]. The studies by Matthias Akamen et al. (2013) on the economic burden of BC in Sweden [35], Katia Cilini et al. (2012) on the economic burden of BC (a systematic review) [19], Alan H. Young et al. (2011) on the economic burden of BC in England [34], and Laura J. Fisher et al. (2007) on the direct medical costs of BC in Australia [36]were consistent with the present study in terms of the largest share of DMC. However, in a systematic review by Leona Besonova et al. (2020) on the economic burden of BC, it was concluded that the largest share of DMC was related to frequent psychiatric interventions [37], the reason for which might be the difference in the prices of psychiatric services in America and the difference in their treatment guidelines. Hence, the present study’s observations differ from previous similar studies conducted in developed nations such as the US.

According to the findings of this study, DNMC accounted for 6.37% of the costs, of which the largest share was that of patient companions’ travel expenses (47.92%). The severalty of companions and the large number of their visits to medical centers for following up the patients’ treatment during hospitalization and discharge could be the reason for the high-cost patient companions’ travels in this research. The results of this study are consistent with the findings of Khosro Keshavarz et al. (2022) on the economic burden of major depressive disorder in Iran [25]. However, our results are not in line with the results of the study by Martin Cloutier et al. (2018) on the economic burden of bipolar disorder in the United States, in which BC drug abuse accounted for the largest share of DNMC [12]. Due to the lack of reliable and community-based information about the costs of crime and drug abuse related to mental disorders and bipolar disease, these costs were not included in the present research.

As far as IC was concerned (15.97%), the highest cost per patient was related to the patients’ work productivity lost due to their absence from work (84.31%). The results of this study were consistent with the studies by Khosro Keshavarz et al. (2022) on the economic burden of major depressive disorder in Iran [25], Matthias Akamen et al. [35], and Martin Cloutier et al. However, the results of the study by Judith Simon et al. (2021) on the costs of bipolar disorder in England [11] showed that productivity lost due to informal care accounted for the largest share of indirect costs, which is inconsistent with the results of the present study. The main reason for this discrepancy could be the high average number of hospitalization days (25.05 days) in the current study; thus; productivity lost due to the patients’ absenteeism was more than productivity lost due to their companions’ absence from work. Khosro Keshavarz et al. (2020) in Iran [25], estimated the average total costs per major depression patient to be $2026, which is almost half of the same amount in our study. The reason for this difference is that the average number of hospitalization days in the aforementioned study is less than in our study, and this in turn affects direct medical costs, non-medical direct costs, and indirect costs.

One limitation of the current research was the forgetting of some costs by the patients and as a result, the approximate inclusion of some costs. In addition, some patients refused to participate in the research. The patients who were referred as outpatients were not included in the study due to the lack of medical records. There were also some deficiencies in the medical information of some patients, including lack of data on comorbidities such as substance abuse. In addition, intangible costs were not calculated due to the impossibility of accurately measuring them. Finally, the lack of domestic research on the economic burden of BD was one of the limitations of this study.

## Conclusion

In general, the results showed that due to the high prevalence of BD in Iran and the chronicity of the disease as well as the need for lifelong treatment, the costs of treating BD could impose a heavy economic burden on the society, the health care system, the insurance system, and the patients themselves. According to the obtained results, and in order to reduce the economic burden of this disease, it is suggested that necessary plans and measures be taken. Considering the high costs of hoteling and ordinary beds, it is suggested that periodic follow-ups of these patients, along with psychological training and counseling by psychologists, be applied to reduce the hospitalization of these patients and consequently, reduce the economic burden of the disease. In addition, to reduce the costs, proper and fair distribution of psychiatrists and psychiatric beds and expansion of home care services are recommended. Following up the treatment of BD patients, especially in future visits, and possibly using the technologies related to the Internet and remote communication channels, i.e. telemedicine, can help reduce the costs of BD patients and their families due to going to the medical centers.

## Data Availability

No datasets were generated or analysed during the current study.
